# Perivascular Epithelioid Cell Tumor of the Ileum Presenting as Diverticulitis

**DOI:** 10.1155/2012/476941

**Published:** 2012-04-29

**Authors:** Saime Unluoglu, Umit Bayol, Nilay Korkmaz, Bekir Ozenen, Fuat Ipekci, Emel Ebru Pala

**Affiliations:** ^1^Department of Pathology, Tepecik Research and Training Hospital, Izmir, Turkey; ^2^Department of General Surgery, Tepecik Research and Training Hospital, Izmir, Turkey

## Abstract

Perivascular epithelioid cell tumors (PEComas) are a group of rare mesenchymal neoplasms. Gastrointestinal PEComas are exceptionally rare, there being only a few case reports in the literature involving the colon and small intestine. Nearly all PEComas show immunoreactivity for both melanocytic (HMB45 and/or Melan-A) and smooth muscle (actin and/or desmin) markers. A 36-year-old male was admitted to the hospital with acut- abdomen. At laparatomy, a nodular mass protruding from the ileum which clinically simulated a diverticulitis was noticed. Gross examination of the specimen revealed a 2 × 1,5 × 1 cm secondarily ulcerated, solid, nodular, gray white tumor mass in the ileal wall. Histologically, tumor cells were composed of nests of round-polygonal epithelioid cells with abundant clear to slightly eosinophilic granular cytoplasm and round vesicular nuclei. The nests were separated by thin fibrovascular septa. Minimal necrosis and low mitotic activity were noticed in the tumor. Immunohistochemically, tumor cells were positive for SMA, HMB45, and Melan-A and negative for CD10, RCC, CD45, CD117, CD34, EMA, and Desmin. Diagnosis was PEComa of the ileum. We report the case of ileal PEComa to remind the unusual presentation (diverticulitis) of these tumors, besides rarity and diagnostic difficulties.

## 1. Introduction

Neoplasms with perivascular epithelioid cell differentiation (PEComas) are a group of rare mesenchymal tumors composed of histologically and immunohistochemically distinctive perivascular epithelioid cells (PECs). The PEC is characterized by positivity with melanocytic (HMB-45 and/or Melan-A) and myogenic (actin and/or desmin) markers [1–7].

PEComa have been reported at diverse anatomic sites, including uterus, ligamentum teres, broad ligament, vagina, vulva, large and small bowel, pelvic side wall, thigh, heart, pancreas, kidney, skins and soft tissues [1, 4–6]. Gastrointestinal PEComas are exceptionally rare, there being only a few case reports in the literature involving the colon and small intestine. Though most of the gastrointestinal PEComa present with abdominal masses, only a few of them with acute abdomen as diverticulitis [4–6].

Here we describe a case of PEComa restricted in the ileum presenting as diverticulitis.

## 2. Case Report

**Figure 1:**
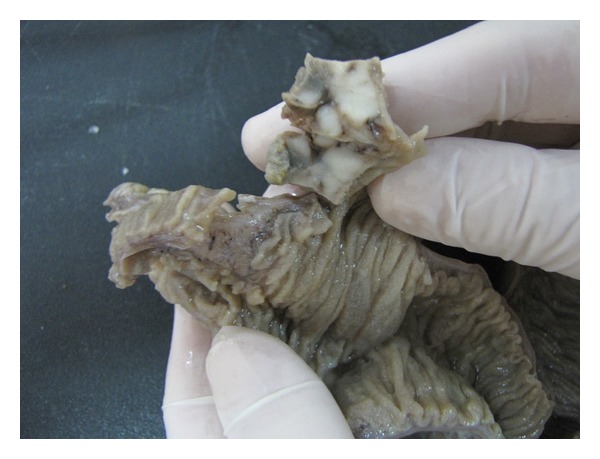
Grossly secondarily ulcerated solid nodular gray white tumor mass.

**Figure 2:**
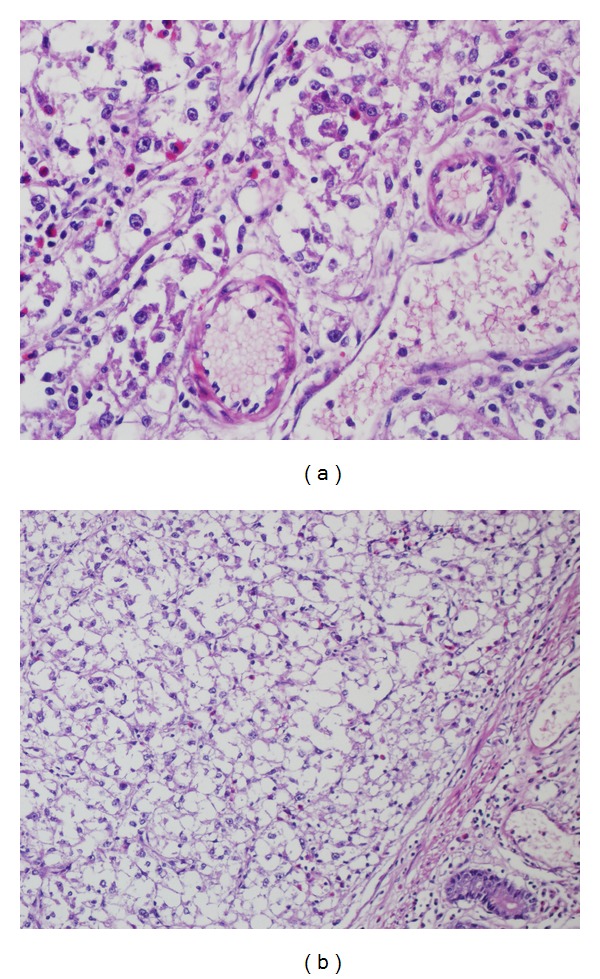
(a) Perivascular tumor cells with clear to slightly eosinophilic granular cytoplasm and round vesicular nuclei, (b) submucosal tumor mass.

A 36-year-old male was admitted to the hospital with acute abdomen. He had abdominal pain for three weeks and it had increased with vomiting in the last 3 days. At laparatomy, abdominal cavity was full of purulent exudate. Carefull exploration of intestinal segments revealed perforation of the ileum and it was attached to the serosal side of descending colon with a nodular mass protruding from the serosal surface of the ileum, simulating a diverticulitis. The terminal 30 cm segment of the ileum containing the diverticula-like mass and appendix vermiformis were resected. Abscess was drained and double ileostomy (one on the right, and one on the left) was performed. Grossly serosal surfaces of the resected ileal segments and appendix were covered with purulent exudate. On the opening of the ileal lumen, a 2 × 1,5 × 1 cm, secondarily ulcerated, solid, nodular, gray white tumor mass was seen close to the resection margin (Figure 1). Four reactive regional lymph nodes were dissected. Histological evaluation demonstrated a tumoral tissue composing nests of round-polygonal epithelioid cells with abundant clear to slightly eosinophilic granular cytoplasm and round vesicular nuclei, located at the periphery of the thin vascular network (Figure 2(a)).

**Figure 3:**
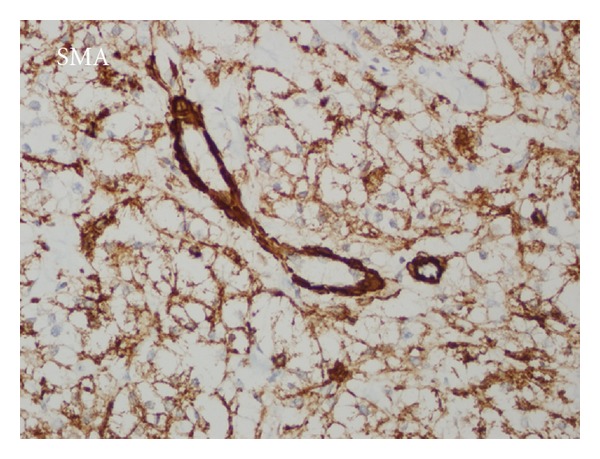
SMA positivity in perivascular tumor cells.

**Figure 4:**
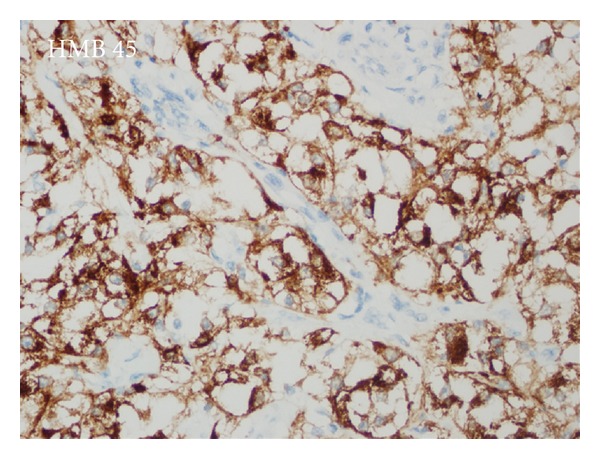
Cytoplasmic HMB45 positivity in perivascular tumor cells.

**Figure 5:**
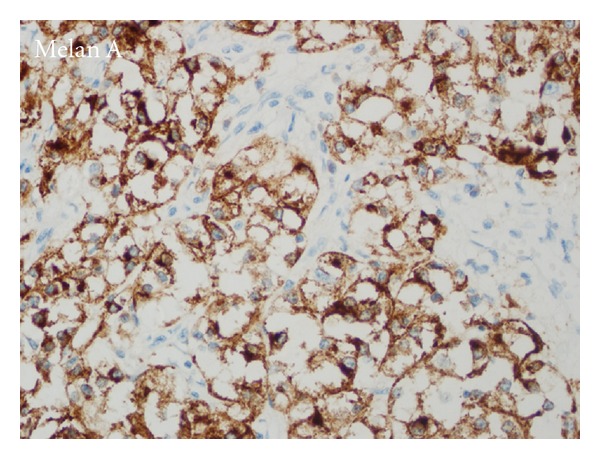
Melan-A positivity in perivascular tumor cells.

 Tumor was secondarily ulcerated and invading submucosa (Figure 2(b)), muscularis propria, and serosa. Minimal necrosis, low nuclear grade (GI), low mitotic activity (1-2/50HPF), and low proliferative index (Ki67 3-4%) were noted. Tumor cells were positive for SMA (Figure 3), HMB45 (Figure 4), Melan-A (Figure 5), CD99, and E cadherin and negative for S100, desmin, CD117, CD34, CD31, AE1/3, EMA, CD10, RCC, CD45, chromogranin A, synaptophysin, ER, PR, cerbB2, cyclin D1, CK20, CK5-6, CK19, bcl2, GCDFP15, MFG, and TTF1 immunohistochemically. So the diagnosis was ileal PEComa.

Postoperative screening endoscopy, contrasted bowel radiography, chest and abdominal CT, PET, bone scan, biochemical analyses were all negative. The patient was discharged. Explorative laparatomy on the third month of the initial operation evaluated no residual tumor confirmed by frozen sections, but only simple postoperative benign granulation reactions. So the stomas were repaired by end to end anastomoses.

The patient did not receive any further treatment and he is well with no evidence of disease at 10th postoperative month.

## 3. Discussion

 Bonetti was the first to suggest the descriptive term perivascular epithelioid cell (PEC) to these unique cells in 1992 [8]. The term “PEComa” was coined by Zamboni in 1996 to describe this rare family of lesions [9]. In 2002, the World Health Organisation (WHO) accepted the designation PEComa as a distinct mesenchymal neoplasm, composed of histologically and immunohistochemically unique PECs [3, 5, 7]. The PEComa family of tumors include renal angiomyolipoma, pulmonary clear cell “sugar” tumor, lymphangiomyomatosis, clear cell myomelanocytic tumor of the falciform ligament, and other rare clear cell tumors of the pancreas, rectum, ileum, abdominal serosa, uterus, vulva, thigh, heart, and so forth [1–5]. A marked female predominance (7/1: female/male) and wide age range (6–75) were noted [2, 5]. Our case was 36-year-old male. Clinically most of the gastrointestinal PEComas present themselves with abdominal mass and pain. Only a few of them present as complicated diverticulitis and attended with acute abdominal features as it had been in the case we present [4].

The differential diagnosis of gastrointestinal PEComa includes epithelioid smooth muscle cell tumor, epithelioid gastrointestinal stromal tumor (GIST), metastatic renal cell carcinoma, metastatic malignant melanoma, metastatic alveolar soft part sarcoma (ASCP), paraganglioma, and anaplastic large cell lymphoma [3, 4, 6]. Epithelioid smooth muscle tumors usually have an eosinophilic cytoplasm and are usually negative for melanocytic markers like HMB-45 and Melan-A [3]. Gastrointestinal stromal tumor (GIST) can be excluded with the negativity for CD117 and CD34 [3]. In our case with the positivity of HMB45 and Melan-A, we excluded epitheliod leiomyoma, with the negativity of CD117 and CD34, GIST. Metastatic renal cell carcinoma (RCC), especially of the chromophobe cell type, is an important differential diagnosis of PEComa. Both of them have epithelioid features and abundant vasculature. RCC is always positive for epithelial markers such as AE1/3, EMA besides vimentin and both CD10, RCC antigen are specific for the diagnosis [3]. Both CD10 and RCC antigen were negative in the case we present.

A diffuse pattern of HMB-45 expression, especially if associated with strong expression of S100 and vimentin in a frankly malignant neoplasm, should prompt consideration of metastatic malignant melanoma. Furthermore, expression of myogenic markers has been very rarely documented in malignant melanoma [4]. In our case S100 was negative and SMA positivity accompanied melanocytic markers. HMB45 reactivity is highly variable in PEComas, ranging from strong and diffuse to focal, but it is characteristically perivascular [4, 6] as in our case.

Metastatic ASPS of the gastrointestinal tract may also be characterized by prominent nucleoli and clear cytoplasm may closely mimic PEComa, but ASPS is always negative for HMB-45 [3]. Paraganglioma may rarely occur in the gastrointestinal tract, but the more organoid growth pattern, the positivity for synaptophysin, chromogranin A and the negativity for HMB-45 and SMA should lead to the correct diagnosis [3]. Neuroendocrine markers were negative and both myogenic and melanotic markers were positive in our case, so we easily excluded ASPS and paraganglioma. With the negativity for CD45, lymphoma is excluded.

 As regarding monoclonal CD99, MIC2 gene product immunoreactivity of our case, it is not surprising since at least 50% (2/4) of the published cases are positive for CD 99. There is no complimentary explanation of such a reaction and the clinicopathologic significance of the immunoreactivity status of CD99 is not known yet [6, 10–12].

Limited literature data has not noted E-cadherin positivity for PEComas, though as an adhesion molecule. E-cadherin immunoreactivity has been noted in almost 100% of malignant melanomas. Melanocytic features of PEComa may be the explanation for E-cadherin immunoreactivity [13, 14]. Both CD 99 and E-cadherin immunoreactivity of PEComas seem noteworthy for future research.

Little is known about the natural history and prognosis of PEComas, as only case reports have been published till now. It appears that PEComas are generally of low grade malignancy, and the benefit of chemotherapy or radiation for nonmetastatic disease has not yet been established. Surgical resection of the tumor in the gastrointestinal tract with the adjacent regional lymph nodes is the mainstay of treatment of the primary tumor and of local recurrences. Nevertheless, malignant PEComa can be a very aggressive disease leading to distant metastases and death. Close clinical surveillance accompanied by imaging and colonoscopy is essential for long-term survival because of the potential for local recurrence and distant metastatic disease [5].

Folpe and colleagues proposed criteria for classifying PEComas into 3 categories: benign, uncertain malignant potential, and malignant. They observed a significant association between tumor size (larger than 5 cm), infiltrative growth pattern, high nuclear grade, necrosis, high mitotic activity and subsequent aggressive clinical behavior. They therefore suggested that PEComas with two or more of these worrisome histological features should be considered malignant [2, 15]. In Folpe's point of view, though it had an infiltrative growth pattern, the tumor was small (2 × 1,5 × 1 cm) with minimal necrosis, low grade, low mitotic activity, low proliferative index; in our case, it can be considered as uncertain malignant potential. As there is no residual tumor mass and as the patient has no sign of disease in the 10th postoperative month, we preferred close clinical surveillance accompanied by imaging and colonoscopy.

In conclusion, we report the case of ileal PEComa to remind the unusual presentation (diverticulitis) of these tumors, besides rarity and diagnostic difficulties.
